# Physiological and Biochemical Responses of Orange Trees to Different Deficit Irrigation Regimes

**DOI:** 10.3390/plants8100423

**Published:** 2019-10-17

**Authors:** Ivana Puglisi, Elisabetta Nicolosi, Daniela Vanella, Angela Roberta Lo Piero, Fiorella Stagno, Daniela Saitta, Giancarlo Roccuzzo, Simona Consoli, Andrea Baglieri

**Affiliations:** 1 Dipartimento di Agricoltura, Alimentazione e Ambiente (Di3A), Università di Catania, Via S. Sofia 98, 95123 Catania, Italy; ipuglisi@unict.it (I.P.); enicolo@unict.it (E.N.); rlopiero@unict.it (A.R.L.P.); d.saitta@unict.it (D.S.); simona.consoli@unict.it (S.C.); abaglie@unict.it (A.B.); 2 Consiglio per la Ricerca in Agricoltura e L’analisi Dell’economia Agraria, Centro di Ricerca Olivicoltura, Frutticoltura e Agrumicoltura, via la Canapona, 1bis - 47121 Forlì, Italy; fiorella.stagno@crea.gov.it (F.S.); giancarlo.roccuzzo@crea.gov.it (G.R.)

**Keywords:** *Citrus* orchards, crop physiology, irrigation, geophysical surveys, water deficit

## Abstract

The article presents the results of research consisting of the application of deficit irrigation (DI) criteria, combined with the adoption of micro-irrigation methods, on orange orchards (*Citrus sinensis* (L.) Osbeck) in Sicily (Italy) during the irrigation season of 2015. Regulated deficit irrigation (RDI, T3) and partial root-zone drying (PRD, T4) strategies were compared with full irrigation (T1) and sustained deficit irrigation (SDI, T2) treatments in terms of physiological, biochemical, and productive crop response. A geophysical survey (electrical resistivity tomography, ERT) was carried out to identify a link between the percentages of drying soil volume in T4 with leaves abscisic acid (ABA) signal. Results highlight that the orange trees physiological response to water stress conditions did not show particular differences among the different irrigation treatments, not inducing detrimental effects on crop production features. ABA levels in leaves were rather constant in all the treatments, except in T4 during late irrigation season. ERT technique identified that prolonged drying cycles during alternate PRD exposed more roots to severe soil drying, thus increasing leaf ABA accumulation.

## 1. Introduction

*Citrus* species are among the most important tree crops for the Mediterranean agricultural sector. Increasing water use efficiency associated with improved irrigation strategies is a priority for these groves to maintain market competitiveness [1]. Applied research has already proved that the application of deficit irrigation (DI) strategies for *Citrus* cultivation is effective and sustainable by maximizing water saving without affecting crop yield [2] and quality parameters [3,4,5,6,7,8]. Nevertheless, it is necessary to quantify the changes in biochemical mechanisms induced by the application of moderate/severe water deficit conditions by analyzing the role of abscisic acid (ABA) [9] and proline accumulations [10,11]. In fact, ABA is recognized as an important stress-signaling hormone, acting in the regulation of stomatal closure, synthesis of compatible osmolytes, and in the upregulation of genes leading to adaptive responses [9]. Among osmolytes, proline is considered an active molecule and its accumulation is putatively mediated by free radicals produced as a result of oxidative stress [10,11]. Proline accumulation plays a protective role for plants in the face of different environmental stresses [12,13]. In *Citrus* trees, proline accumulation is generally associated with water loss induced by soil water depletion, elevated transpiration rates associated with high temperatures, and with different basal levels of proline between genotypes and cultivar [14,15].

With respect to the existing literature, the innovative aspects introduced in this study concern the investigation of the effects of DI strategies application on biochemical and physiological responses of mature *Citrus sinensis* (L.) Osbeck crops. The specific objectives of the study are: (i) To assess the sustainability of partial root-zone drying (PRD), regulated deficit irrigation (RDI), and sustained deficit irrigation (SDI), integrated with surface and sub-surface micro-irrigation techniques, in maintaining adequate physiological and productive crop responses; and (ii) to identify links between the quota of water deficit and physiological and biochemical plant responses.

## 2. Results

### 2.1. Soil Characteristics and Plant Physiological Response to Deficit Irrigation Strategies

**Figure 1:**
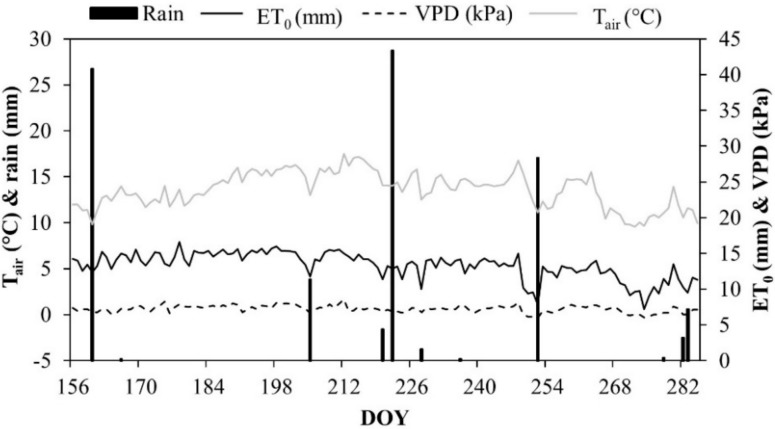
Air temperature (T_air_), vapor pressure deficit (VPD), reference ET (ET_0_), and rainfall at the experimental site during the irrigation season 2015.

Climate conditions during 2015 were typical of Mediterranean semi-arid regions, with hot and dry summers. During the irrigation season (June to October 2015), the maximum air temperature (T_air_) reached 30 °C, with mean values of vapor pressure deficit (VPD) and relative humidity (RH) equal to 0.62 kPa (Figure 1) and 69.8%, respectively.

The reference evapotranspiration (ET_0_) had a mean of 5.4 mm d^−1^, reaching a total of around 700 mm. ET_c_ was about 300 mm and rainfall was less than 100 mm. The amount of irrigation (mm) supplied at the different treatments and the corresponding water savings are reported in Table 1.

The water deficit (%), shown in Table 1, was only slightly different from those theoretical fixed for each treatment, thus demonstrating the adequacy of the irrigation system setup. Table 2 reports the main physical and chemical soil characteristics for each of the different treatments. Cation exchange capacity (CEC), electrical conductivity (EC), and pH did not show any differences among the treatments.

Soil in T4 (PRD) showed lower organic carbon (OC) content than the other treatments, whereas T3 (RDI) showed higher N_TOT_ values. The highest available phosphate content (P) was observed in T1 and the lowest in T4.

**Figure 2:**
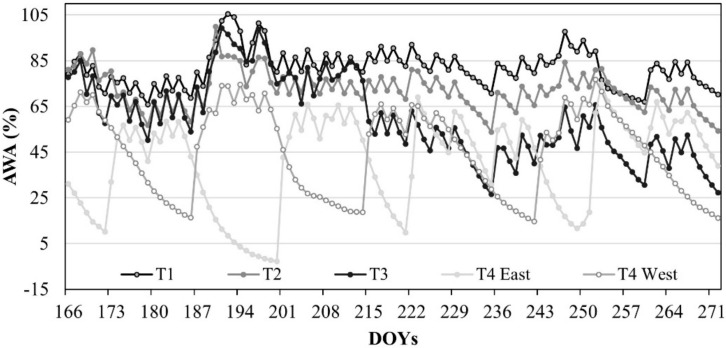
Seasonal evolution of the amount of available water (AWA) in the soil profile of the DI treatments. DOY: Day of the year.

The amount of available water (AWA) in the investigated soil profile was maintained around 80% in T1, 71% in T2, 58% in T3, 43% in T4 East, and 46% in T4 West (Figure 2).

**Figure 3:**
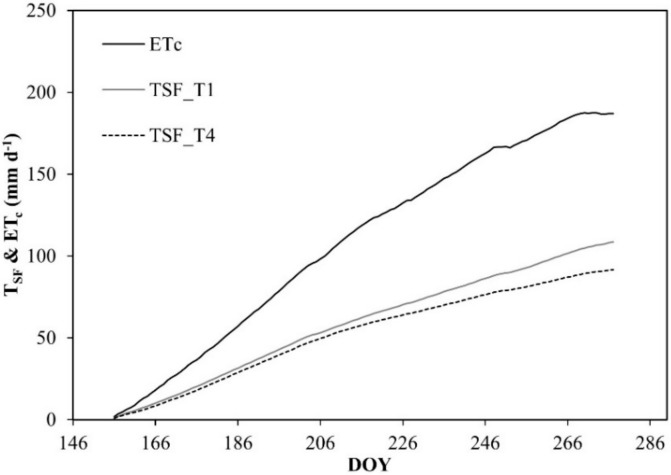
Cumulative values of T_SF_ and ET_c_ rates in T1 and T4. DOY: Day of the year.

In T1, T2, and T3, the soil volumetric water content (θ_v_) remained very close to field capacity (θ_FC_) condition, while θ_v_ in T4 was characterized by the expected alternation between drying and wetting cycles (T4, East and West), decreasing slightly below the threshold of the wilting point (θ_WP_) (minimum θ_v_ value of 0.11 cm^3^ cm^−3^). Figure 3 reports the cumulative plant transpiration (T_SF_) for T1 and T4, obtained with heat pulse method (HPV) method.

Results evidenced that T4 transpired 92 mm during the irrigation season, about 58% less than ET_c_, while T1 transpired about 49% less than ET_c_. The discrepancy between T_SF_ rate in T1 and crop evapotranspiration (ET_c_) was mainly due to soil evaporation. As reported in Table 3, fresh and dry weights of the leaves were similar among the different irrigation treatments during the monitoring.

As an exception, a slight increase of the T2 indicators compared to T1 was detected at the beginning of the irrigation season and at the end of September (day of the year (DOY) 272). The open stomata were fairly constant, while the total stomata decreased slightly in T2. Leaf are index (LAI) and photosynthetically active radiation (PAR) were fairly similar among the different treatments during the monitoring, with the exception of a certain reduction after DOY 174, due to pruning. Total yield and equatorial section (ED) did not show any difference among the DI treatments and T1 (Table 4).

**Figure 4:**
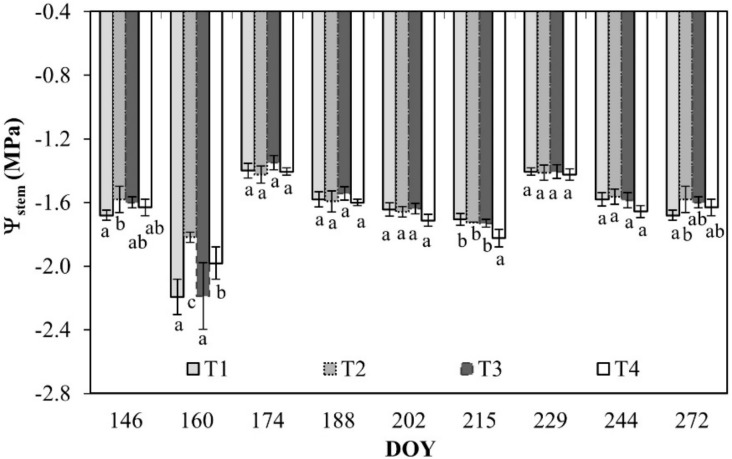
Stem water potential (Ψ_stem_) for leaves of orange trees at the different irrigation treatments. Vertical bars are standard deviation. For each date, the average values that share a letter are not statistically different. DOY: Day of the year.

The values of stem water potential (Ψ_stem_) resulted fairly similar during the monitoring and among the different treatments. The lowest values (–1.8/–2.2 MPa) were recorded at DOY 160, before the beginning of the irrigation season (DOY 166 to DOY 289). In all the treatments, Ψ_stem_ ranged between –1.4 and –2.2 MPa (Figure 4).

**Figure 5:**
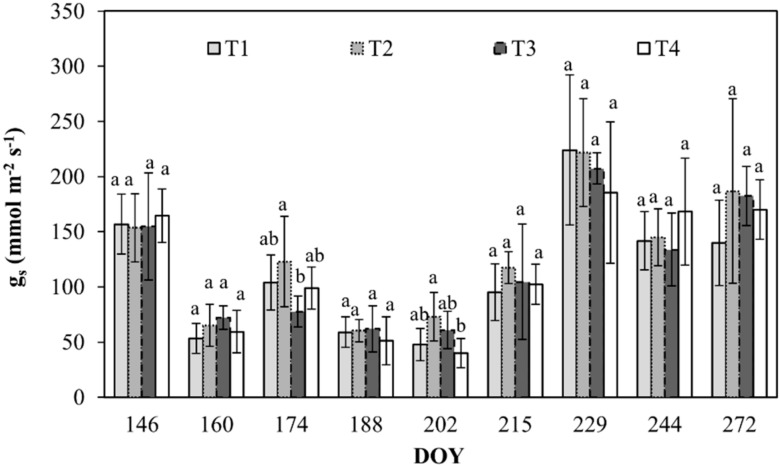
Stomatal conductance (g_s_) for sunny leaves of orange trees at the different irrigation treatments. Vertical bars are standard deviation. For each date, the average values that share a letter are not statistically different. DOY: Day of the year.

During the monitoring, g_s_ varied from a maximum of about 224 mmol m^−2^ s^−1^ in T1 to a minimum of 40 mmol m^−2^ s^−1^ in T4 (Figure 5).

### 2.2. ABA and Proline Detection in Orange Leaves

**Figure 6:**
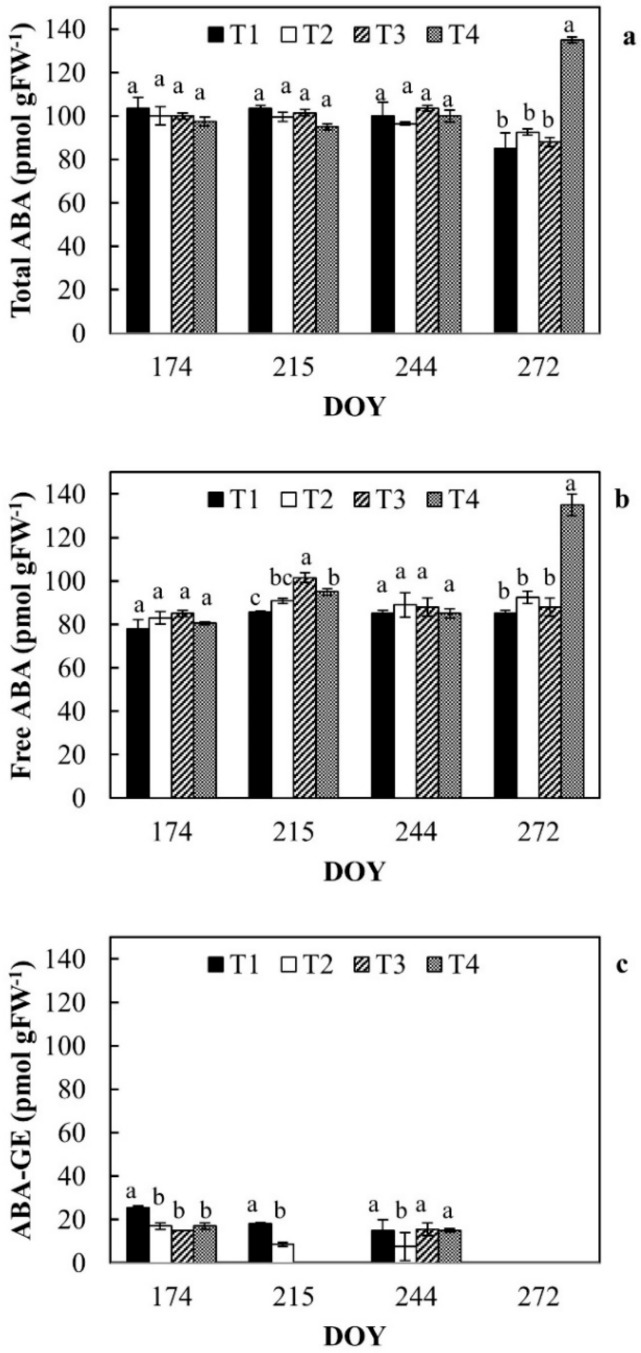
Total ABA (**a**), free ABA (**b**) and conjugated ABA (**c**) in leaves of orange trees at the different irrigation treatments. For each date, the average values that share a letter are not statistically different. DOY: Day of the year.

Total ABA, free ABA, and ABA-GE contents in leaves are reported in Figure 6a–c, respectively.

During DOY 272 (late irrigation season), a sharp increase in free ABA was registered in T4 (PRD). During the monitoring period, ABA components did not show differences among the irrigation treatments; only during DOY 215 free ABA of RDI leaves (T3) increased compared to the other treatments (Figure 6b). ABA-GE was generally higher in T1 (Figure 6c) and at DOY 215 it was not detected in treatments T3 and T4. At the end of the irrigation season, in all the investigated treatments, ABA-GE was completely hydrolyzed into free ABA to contrast water stress conditions.

**Figure 7:**
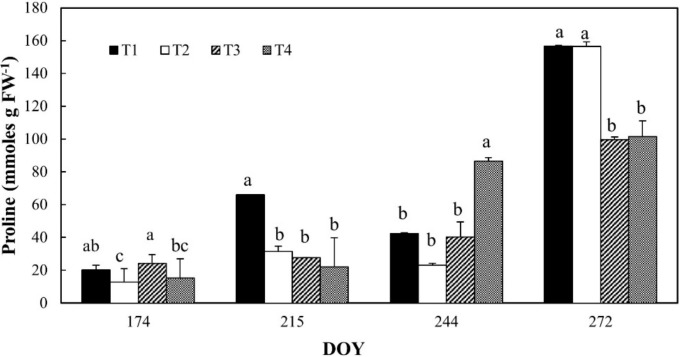
Proline content in leaves of orange trees at the different irrigation treatments. For each date, the average values that share a letter are not statistically different. DOY: Day of the year.

Proline content in leaves of all the irrigation treatments increased during the monitoring period, reaching a maximum at DOY 272 (Figure 7) for T1 and T2. At the end of irrigation season, T1 and T2 treatments increased their proline content, respectively, 3.7-fold and 6.8-fold compared to previous values (DOY 244). At DOY 215, T1 resulted to be higher than other treatments, whereas at DOY 244, T4 showed values of proline around twice as high than others.

### 2.3. The Use of ERT to Identify Soil Drying Pattern under PRD

**Figure 8:**
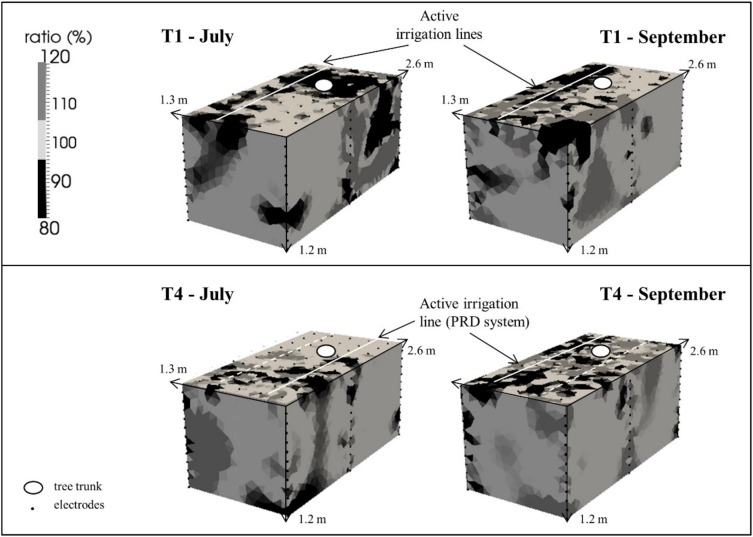
Electrical resistivity (ER) changes on the investigated soil volumes in T1 and T4.

Figure 8 shows the % of increase (ration higher than 100%) or decrease (ratio lower than 100%) of electrical resistivity (ER) in the investigated soil volumes in T1 and T4, during the electrical resistivity tomography (ERT) surveys, compared to the initial conditions (background, ratio of 100%), when no irrigation was applied.

**Figure 9:**
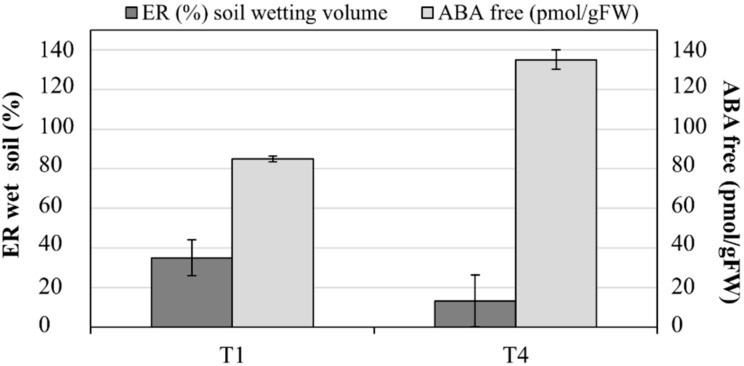
Free abscisic acid (ABA) accumulation in leaves (pmol gFW^−1^) and % of wetted soil by electrical resistivity tomography (ERT) in T1 and T4.

The main effects of the simultaneous phenomena occurring within the soil of T1 and T4 (i.e., infiltration and root water uptake) were wetting (ER decrease) and drying (ER increase) patterns. At the end of the irrigation phase, about 35% (± 9.1%) of the soil volume in T1 presented a marked decrease in the ER due to the progression of the infiltrated irrigation front. At the same time, on average, 13% (± 13%) of soil volume in T4 was increased by wetting patterns, with a decrease in ER. Recognizable soil drying pattern, corresponding to an increase in ER values, interested on average more than 25% (± 10%) of the soil volume in T4 and less than 15% (± 5%) of the soil volume in T1. Figure 9 shows the comparison between free ABA accumulation and the % of decreasing ER in the soil volumes in T1 and T4.

## 3. Discussion

The results obtained during the research encourage the adoption of DI criteria for high-value Mediterranean crops, like orange orchards, particularly susceptible to the occurrence of climatic change scenarios. This study confirms and builds upon previous research carried out on the same issue by the same authors in the same study area [6,7,16]. Additionally, it introduces new observations while identifying new possibilities of irrigation for the minimization of the negative effects of severe water deficit (i.e., 50% of ET_c_ in T4).

First of all, this study shows that it is possible to maintain soil fertility even in severe deficit irrigated treatments, as already shown in different works [17]. As a matter of fact, despite its lower OC with constant and equilibrate N_TOT_ [18], T4 sustained yield. This can be explained by previous studies, which demonstrated that the effects on soil physical–chemical composition affecting root water uptake, under severe and prolonged water deficit conditions but frequent soil rewetting, allow partial compensation of induced side effects [19,20].

Second, the study provides useful information on the various irrigation methods, in particular on surface drip irrigation vs. sub-surface drip irrigation, or T1 vs. T2. The SDI (T2) treatment, by eliminating about 25% of water losses for evaporation, is quite similar to T1. As suggested by Consoli et al. [6,7] and García-Tejero et al. [5], the plants in T1 and T2 had similar physiological, biochemical, and productive responses.

Third, the study gives useful information about the physiological response (i.e., Ψ_stem_, g_s_, and stomata open/closure) of the deficit irrigated orange orchards. The physiological indicators are very sensitive in these kinds of studies; the response to deficit conditions is snap and show coherence between stem water potential and stomata conductance. As expected, the most negative Ψ_stem_ of about –1.8 MPa was recorded for T4 in the middle of the irrigation season. Generally, values of Ψ_stem_ and g_s_ are consistent with moderate water stress conditions, as confirmed by several studies [7,21,22,23]. Opened stomata (%) as well as total stomata (number) were not influenced by water deficit conditions (Table 3), although Xu and Zhou [24] and Damour et al. [23] found a certain degree of leaf trait plasticity (i.e., determining adaptation) in response to environmental changes, including water stress conditions [25]. Plants in T4 acquired a stomatal control mechanism and lower water content in the soil that allowed them to regulate the T_SF_ mechanism on the released water availability (T_SF_ in T4 is about 15% less than in T1).

The synchronization of stomatal resistance and Ψ_stem_ may occur due to a hydro-active, negative feedback response, involving a biochemical- (e.g., proline) and hormonal- (e.g., ABA) mediated response of guard cells to perturbations of the leaf water potential or hydro-passive [26]. In our case, the Ψ_stem_ changes were not associated with proline accumulation, suggesting the hypothesis of a physiologic accumulation of this osmolite, as confirmed by the literature [27,28,29,30,31]. Some authors have shown that the proline accumulation can occur in physiological conditions related to growth purposes, since a significant amount of this amino acid increased its concentration in the reproductive organs of different non-stressed plant species [31,32,33]. This is in line with the results obtained in this study in T1 and T2 treatments of DOY 272.

Fourth, this study involved an interesting aspect regarding the ABA contents found in leaves of the different irrigation treatments. Generally, in experimental open-field conditions like ours, numerous abiotic stress conditions co-occur simultaneously, producing a unique plant response. For example, as suggested by Zandalinas et al. [14], while water stress could induce ABA accumulation in citrus tissues, heat stress may inhibit ABA accumulation; thus, stressed citrus leaves may undergo substantially different programs regulating ABA homeostasis.

Under water stress conditions, apoplastic pH increases resulting in greater retention of ABA, functioning as a signal to reduce transpiration in leaves [34]. Endogenous free ABA levels are regulated through the coordinated action of biosynthesis, catabolism, and conjugation that mainly produces ABA-GE, which is considered one of the major inactive forms of ABA [34,35]. Recently, Romero et al. [36] found that in *Citrus sinensis* L. Osbeck, the response to moderate dehydration in ABA-deficient mutant included both ABA-dependent and independent pathways. Accordingly, our results support the hypothesis that, in orange trees, an ABA-independent pathway regulates the stress response in field, where different stressing factors along with water deficit irrigation occur.

Moreover, our results showed that, with the exception of PRD (T4), all the investigated DI treatments, if compared to the control, do not induce any ABA stress signaling involved in an adaptive response. The effect of this increase of ABA levels at T4 can putatively trigger a later adaptive response, as suggested by Romero et al. [36]. In fact, at the end of September, plants reset their ABA-GE reserve, making it all available.

Finally, recent studies indicate that prolonging the drying cycles during alternate PRD exposes more roots to severe soil drying, increasing root and leaf ABA accumulation, and enhancing crop yields and quality [8]. As in our study case, leaf ABA accumulation was registered in the DI treatment, which substantially decreased the transpiration rate (PRD transpires about 44% less than ET_c_) [37]. These observations are consistent with a model that explains leaf free-ABA concentration of PRD plants as a function of xylem ABA concentrations emanating from the irrigated and drying parts of the root system and the relative sap flow from each plant [38,39]. Moreover, in our study, an inverse relationship was observed between free ABA concentrations and soil wetting dynamics by ERT (Figure 9), confirming that, as evidenced by Pérez-Pérez et al. [8], prolonged exposure of half of the root system to drying soil combined with alternate re-watering can improve ABA accumulation (169.96 ± 24.83 pmol gFW^−1^ at the end of the irrigation season in T4).

## 4. Materials and Methods

### 4.1. Experimental Site, Climatic Data, and Crop Water Demands

**Figure 10:**
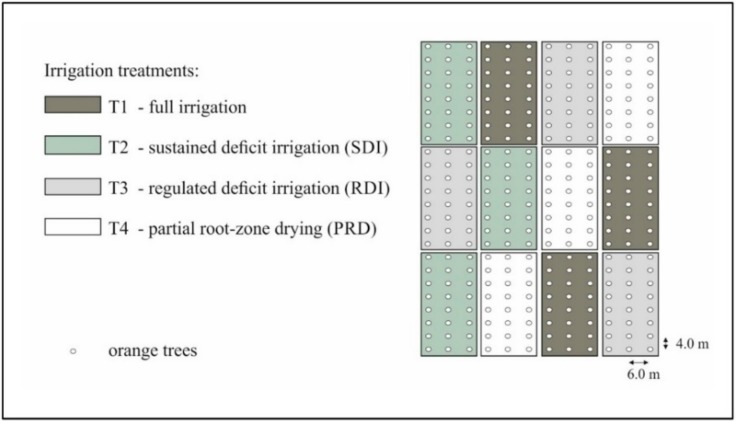
Lay-out of the deficit irrigation (DI) experimental site (Eastern Sicily, Italy).

The study was carried out in a 1 ha experimental field in Eastern Sicily, Italy (latitude 37°20′ N, longitude 14°53′ E; 50 m altitude) where orange trees *cv* Tarocco Sciara grafted on Carrizo citrange, (*Poncirus trifoliata* (L.) Raf. × *C. sinensis* (L.) Osbeck) were planted in 2010, with a between-row spacing of 6 m and a within-row spacing of 4 m. The crops have undergone DI regimes since the youth phase (i.e., the year 2010). The experiment was set as a randomized block design with three irrigation treatments, replicated three times [6]: (i) Full irrigation (control, T1), 100% crop evapotranspiration (ET_c_) using a surface drip irrigation system; (ii) sustained deficit irrigation (SDI, T2) irrigated at 75% ET_c_ using a subsurface drip irrigation system; (iii) regulated deficit irrigation (RDI, T3) irrigated at 100% ET_c_, except in II phenological stage (i.e., fruit growth, at 50% ET_c_), using a surface drip irrigation system; and (iv) partial root-zone drying treatment (PRD, T4) irrigated at 50% of ET_c_ where the water was supplied by two drip lines placed respectively at the eastern and western side of the plants, and used alternatively every 14 day intervals. Each treatment consists of three rows of eight trees, for a total of 24 plants (Figure 10).

Irrigation was applied during the irrigation season 2015, from mid-June (DOY 166) to mid-October (DOY 289), three times per week, early in the morning.

An automatic weather station, located at the farm, registered hourly meteorological data (i.e., solar radiation, R_s_, W m^−2^, air temperature, T_air_, °C, relative humidity, RH, %, wind speed, u, ms^−1^, and direction of rainfall), which were then used to calculate reference ET (ET_0_ mm d^−1^) through the Penman–Monteith approach [40,41]. Crop evapotranspiration (ET_c_) was obtained by multiplying daily ET_0_ by the seasonal crop coefficient (K_c_) for orange orchard (i.e., 0.7) as assessed by Consoli et al. [42,43]. Correction coefficients were applied to K_c_ to consider canopy size (i.e., 0.65), irrigation method efficiency (i.e., 0.9), and the occurrence of rainfall events.

Measurements of transpiration (T_SF_) at tree level were obtained by the heat pulse velocity (HPV) technique [44], which is based on the measurement of temperature variations (ΔT) produced by a heat pulse of short duration (1–2 s). The measurements were taken in two temperature probes installed asymmetrically on either side of a linear heater inserted into the trunk. In particular, one 4 cm sap flow probe, with two embedded thermocouples (Tranzflo NZ Ltd., Palmerston North, NZ), was positioned in the trunks of the trees (i.e., at south side of the trunk, 20 cm from the ground) and wired to a data-logger (CR1000, Campbell Sci., Logan, UT, USA) for heat-pulse control and measurement; the sampling interval was set at 30 min. Data were processed according to Green et al. [45] to integrate sap flow velocity over sapwood area (determined as reported in Consoli et al. [7]) and calculate transpiration fluxes.

Irrigation water had electrical conductivity (EC 25 °C) of 2.02 dS m^−1^ (medium salinity) and pH of 7.30. The soil volumetric water content (θ_v_) was measured using 10 ECH_2_O probes (Decagon, Inc., Pullman, WA, USA) located at different depths (0.15–0.40 m) of the irrigation treatments. The amount of available water (AWA) was calculated according to the following equation:

(1) $$\mathrm{AWA}=\frac{\theta_{v}-\theta_{WP}}{\theta_{FC}-\theta_{WP}},$$

where θ_v_ (m^3^ m^−3^) is the actual soil water content, θ_WP_ (m^3^ m^−3^) is the soil water content at the wilting point, and θ_FC_ (m^3^ m^−3^) is the soil water content at the field capacity. The soil at the experimental site resulted fairly uniform, with a sandy-loam texture (69.7% sand, 10.5% clay, 19.8% silt), mean θ_FC_ (pF = 2.5) and θ_WP_ (pF = 4.2) of 24% and 14%, respectively [46,47]. Soil samples were collected at depths between 0.05 and 0.25 m for physical–chemical laboratory determinations, air-dried, and then sieved at 2 mm. Organic carbon (OC), nitrogen (N), cation exchange capacity (CEC), Ca^2+^, Mg^2+^, K^+^ and Na^+^ exchangeable elements, available phosphate (P), electric conductivity, and pH were determined according to Page et al. [48] and following the Italian Ministerial Decree (MD) 13/09/1999.

### 4.2. ABA and Proline Content Detection in Orange Leaves

Trees leaves (1 g) were randomly sampled from the four treatments subjected to different irrigation treatments, frozen in liquid nitrogen, and stored at −80 °C until further laboratory analysis. The abscisic acid (ABA) concentration was determined with a Phytodetek ABA enzyme immunoassay test kit (Agdia, Elkhart, IN, USA), according to the manufacturer’s protocol. The frozen leaves were ground into powder and homogenate in 10 mL of 80% acetone, 0.5 g L^−1^ citric acid, and 20 mg L^−1^ butylated hydroxytoluene [49]. The suspension was centrifuged at 3000 × g for 5 min, and the supernatant was diluted with Tris-buffered saline (45 mM Tris-HCl, pH 7.8, 90 μM MgCl_2_, 0.135 M NaCl, and 3 mM sodium azide). Samples were subdivided into two fractions to determine both free and total ABA. To determine the total ABA, the hydrolysis of ABA glucosyl ester (ABA-GE) was performed by adding 0.1 M sodium hydroxide and incubating in a water bath at 60 °C for 1 h. Then, samples were cooled in an ice bath and pH was adjusted by chlorhydric acid. The absorbances were detected at 405 nm. The ABA concentration was determined from a standard curve. ABA-GE was calculated by subtracting free ABA to total ABA.

Proline was determined spectrophotometrically following the ninhydrin method of Bates et al. [50] modified by Khedr et al. [51]. Briefly, the frozen citrus leaves (1 g) were homogenized in 3% aqueous sulphosalicylic acid and the residues were removed by centrifugation at 12,000× *g* for 10 min. The supernatant (1 mL) was mixed with 1 mL of glacial acetic acid and ninhydrin reagent in a 1:1 (*v*/*v*) ratio. The reaction mixture was incubated at 100 °C for 1 h. After extraction with toluene, the absorbance of the organic phase was read at a wavelength of 520 nm, using toluene as a blank. The proline concentration was determined from a standard curve using D-proline.

### 4.3. Plant Physiological Indicators and Productive Crop Features

Stem water potential (Ψ_s_) was measured at midday with a pressure chamber (SKPM 1405/40, Skye Instruments, Llandrindod Wells, UK), as described by Scholander et al. [52] and following the procedure reported in Turner [53]. For each treatment, two leaves from four different trees of each replica (total of 24 leaves for each treatment) were monitored. Measurements were carried out on fully exposed sunlight leaves, bagged in plastic bags, and covered with silver foil at least 1 h prior to determinations. Stomatal conductance (g_s_, mmol m^−2^ s^−1^) was obtained using a leaf porometer (Decagon Devices Inc., Pullman, WA, USA) during the central hours of the day (between 11:00 and 13:00). Measurements were performed in six fully exposed leaves per tree and four trees per treatment. Leaf stomatal density was determined using the impression approach [54,55], which expresses the number of stomata per unit leaf area (opened and closed stomata). The impression was taken from the surface of around 0.02 m^2^ of fully expanded leaves in the mid-area between the central vein and the leaf edge. The thin film was peeled off from the leaf surface and the number of stomata was counted by using an image analyzer (Leica ASM 68 K) and the software Image Tool. Leaf area index (LAI, m^2^ m^−2^) and photosynthetically active radiation (PAR, %) were monitored at plant level with a ceptometer (Accu PAR LP-80, Decagon Devices Inc., Pullman, WA, USA). Fresh and dry weights (g) were obtained on five leaves per tree per irrigation treatments. Leaf dry weight was obtained by drying leaves in an oven at 75 °C until constant weight was reached.

Total yield (t ha^−1^) and fruit weight (g) were determined at time of commercial harvest in February 2016. Ten fruits were chosen from 12 trees per treatment and replication and were analyzed for determining the equatorial section (ED, mm) through a caliber tape.

### 4.4. The Use of Electrical Resistivity Tomography (ERT) to Identify Soil Drying Pattern under PRD

Soil electrical resistivity (ER) distribution represents an indirect indication of the soil water state (e.g., porosity, water content, and pore water salinity) [7]. Electrical resistivity tomography (ERT—see Binley et al. [55], among others) consists of the injection of an electrical current in the subsoil by a pair of electrodes and the subsequent measurement of the electrical potential. This acquisition is repeated through many combinations of transmitting and receiving electrodes in order to acquire data that can then be inverted to produce two-dimensional (2-D) or three-dimensional (3-D) images of ER distribution on the subsurface. The use of borehole electrodes enhances resolution at depth. In this study, small-scale 3-D ERT monitoring was conducted around 2 selected orange trees irrigated at full level (T1) and by PRD (T4). For each tree, the setup consists of 6 boreholes (1.2 m deep,) each housing 12 electrodes (vertically spaced 0.1 m), plus 48 surface electrodes (spaced 0.26 m on a regular square grid) (details in Vanella et al., [56,57]). The ERT setup covered a soil volume of about 4 m^3^ (1.3 × 2.6 × 1.2 m). The 3-D ERT monitoring was conducted during the mid and at end of the irrigation season 2015 (DOYs 195–264). For each 3-D ERT monitoring, two datasets were acquired using a Syscal Pro Switch 72 resistivity meter (IRIS Instruments, Orléans, France), one related to the initial condition, to be used as background dataset, and the one after the irrigation phase (time-lapse mode). A total of 8 drippers were located at the surface of the control volume in T1 and T4. In T4, irrigation was supplied by the active pipeline located on the east or west sides of the tree trunk and lasted about three hours. Data quality was assessed using a full acquisition of reciprocals to estimate the data error level (see Binley et al. [55], amongst many others). The estimation of the ER as a percentage of the background ER was obtained by 3-D data inversion using the Occam approach as implemented in R3t software package [58].

### 4.5. Statistical Analysis

The acquired data were subjected to one-way analysis of variance (ANOVA) (Statistica 6.0 package, Statsoft Inc., Tulsa, OK, USA). A fixed factor corresponding to the four-level irrigation treatment, T1, T2, T3, and T4 (randomly distributed at the experimental site under study), was used for analyzing the physiological and biochemical responses of orange trees to the different deficit irrigation regimes. In the case of significant difference (*p* value < 0.05), means were separated using the Tukey’s HSD test.

## 5. Conclusions

The challenge for agriculture in the near future will be to combine water use efficiency with increased resilience in all the productive systems. In this view, the study herein presented focuses on the feasibility of the application of moderate (RDI) and severe (PRD) water deficit conditions to high-value cropping systems, like orange orchards, in Mediterranean climatic conditions. The main conclusions that can be pointed out are the following:

- DI strategies (i.e., RDI and PRD) did not alter soil fertility among treatments and compromise the nutrients uptake by plants;
- The sub-surface drip irrigation (SDI) and the control had similar behaviors, but SDI, allowing the reduction of soil evaporation losses, should be preferable to surface drip irrigation;
- The physiological response to water stress conditions did not show particular differences among the irrigation treatments, not inducing detrimental effects on crop production features;
- Proline accumulation in orange leaves results were not related to water deficit conditions; rather, proline reached the highest values in the well-irrigated T1 and T2 treatments;
- ABA levels in leaves were rather constant in all the treatments, except in T4 (PRD) during September; this response might produce a late adaptive crop production response;
- Prolonged drying cycles during alternate PRD exposed more roots to severe soil drying, thus increasing leaf ABA accumulation.

## Figures and Tables

**Table 1:** Irrigation rates and water savings for the different treatments (T1, control; T2, SDI; T3, RDI; T4, PRD) at the experimental site during irrigation season 2015.

	T1	T2	T3	T4
**Irrigation Rates (mm)**	279.0	204.2	174.2	158.0
Water Savings * (%)	--	26.8	37.6	43.4

* (1- (irrigation T_i_ = 2, 3, 4 / irrigation T1)) × 100).

**Table 2:** Chemical and physical soil properties at the different irrigation treatments in 2015.

Treat.	OC (g kg^−1^)	N_TOT_ (g kg^−1^)	CEC (meq 100 g^−1^)	Ca (g kg^−1^)	Mg (g kg^−1^)	K (g kg^−1^)	Na (g kg^−1^)	P_avail_ (mg kg^−1^)	EC (mS cm^−1^)	pH
**T1**	10.8 a	0.6 b	48.9 a	5.2 b	2.9 a	1.2 b	1.8 a	56.0 a	0.32 a	8.1 a
**T2**	10.3 a	0.3 c	43.3 a	7.8 a	1.7 b	1.0 b	0.9 b	42.9 ab	0.26 a	8.3 a
**T3**	12.7 a	1.6 a	46.1 a	5.8 b	2.1 ab	1.9 a	1.3 b	44.9 ab	0.27 a	8.3 a
**T4**	8.8 b	0.8 b	40.0 a	4.9 b	2.7 ab	1.9 a	1.6 a	39.9 b	0.25 a	8.5 a

OC: Organic carbon; CEC: Cation exchange capacity; EC: Electrical conductivity. Values in the same column followed by different letters were significantly different (*p* < 0.05).

**Table 3:** Physiological plant response indicators to DI. Values (average and standard deviation) of the same columns for each DOY followed by different letters were significantly different (*p* < 0.05).

DOY	Treatment	Fresh Weight (g/leaf)	Dry Weight (g/leaf)	Total Stomata (number/m^2^)	Opened Stomata (%)	LAI (m^2^ m^−2^)	PAR (%)
174	T1	0.8 ± 0.2 b	0.3 ± 0.1 b	372.3 ± 52.2	66.6	4.7 ± 0.9	71.5 ± 4.6
	T2	1.1 ± 0.3 a	0.4 ± 0.1 a	369.3 ± 51.0	77.9	5.2 ± 0.3	75.2 ± 2.8
	T3	0.9 ± 0.2 ab	0.3 ± 0.1 ab	358.9 ± 60.8	68.5	5.5 ± 0.8	75.3 ± 2.0
	T4	0.9 ± 0.3 ab	0.3 ± 0.1 ab	382.4 ± 51.2	69.9	5.0 ± 0.7	73.4 ± 3.6
215	T1	1.1 ± 0.3	0.4 ± 0.1	328.8 ± 51.0	36.6	4.0 ± 0.6	69.5 ± 6.3
	T2	1.8 ± 0.4	0.4 ± 0.2	294.6 ± 77.9	31.1	4.3 ± 0.3	74.8 ± 1.7
	T3	1.1 ± 0.2	0.4 ± 0.1	320.1 ± 90.1	39.7	4.5 ± 0.7	79.1 ± 7.1
	T4	1.2 ± 0.2	0.5 ± 0.1	344.4 ± 61.1	38.9	4.9 ± 0.3	80.4 ± 13.3
244	T1	1.2 ± 0.4	0.4 ± 0.14	279.0 ± 50.8 ab	36.8	3.4 ± 0.5	69.1 ± 8.2
	T2	1.1 ± 0.3	0.4 ± 0.14	260.5 ± 54.0 b	24.8	4.1 ± 0.6	74.7 ± 1.3
	T3	1.1 ± 0.3	0.4 ± 0.10	324.3 ± 53.4 a	35.9	3.3 ± 0.7	70 ± 3.8
	T4	1.1 ± 0.2	0.4 ± 0.11	333.0 ± 56.9 a	27.8	3.6 ± 0.7	69.3 ± 9.6
272	T1	1.6 ± 0.3 a	0.6 ± 0.1 a	356.6 ± 39.4 ab	61.5	2.2 ± 0.3	44.7 ± 9.6
	T2	1.2 ± 0.2 b	0.5 ± 0.1 b	354.6 ± 48.5 b	75.5	2.6 ± 0.9	54.4 ± 12.2
	T3	0.9 ± 0.2 b	0.4 ± 0.1 b	405.9 ± 70.7 a	56.5	2.4 ± 0.3	53.7 ± 11.3
	T4	0.9 ± 0.2 b	0.4 ± 0.1 b	397.8 ± 78.8 ab	63.1	2.0 ± 0.5	43.3 ± 6.5

**Table 4:** Plant production characteristics at harvest (February 2016). Values refer to average and standard deviation.

Treatment	Mean Fruit Weight (g)	Total Yield (t ha^−1^)	Equatorial Section (ED mm)
T1	259.3 ( ± 7.6)	24.6 ( ± 1.78)	77.3 ( ± 0.78)
T2	264.8 ( ± 11.1)	22.7 ( ± 1.38)	78.7 ( ± 1.46)
T3	276.0 ( ± 10.4)	23.5 ( ± 2.48)	79.2 ( ± 1.09)
T4	253.0 ( ± 12.0)	24.7 ( ± 3.8)	77.6 ( ± 1.30)
